# The ADHD-200 Consortium: A Model to Advance the Translational Potential of Neuroimaging in Clinical Neuroscience

**DOI:** 10.3389/fnsys.2012.00062

**Published:** 2012-09-05

**Authors:** The ADHD-200 Consortium

Neuropsychiatric imaging remains a pioneering frontier in modern medicine. Recent decades have witnessed marked advances in identifying biological correlates for a broad array of illnesses (Hillary et al., 2007; Ritsner, 2009; Linden and Thome, 2011; Shenton and Turetsky, 2011). However, our understanding of the underlying pathophysiology of neuropsychiatric illnesses remains insufficient (Ecker et al., 2010; Linden, 2012). Equally problematic, translational promises have yet to be delivered, as clinically useful biomarkers are rarely attained (Hyman, 2002; Nestler and Hyman, 2010). As such, psychiatry remains uniquely reliant upon a diagnostic and classification system derived from clusters of symptoms rather than etiology or neurobiology (Hyman, 2007; van Praag, 2008; Nesse and Stein, 2012). Recent works demonstrating the feasibility of predicting maturational and disease status from functional MRI and morphometric imaging data (Craddock et al., 2009; Dosenbach et al., 2010; Ecker et al., 2010) have rekindled hopes for the eventual development of imaging-based tools to inform clinicians in their efforts (Bullmore et al., 2009; Fox and Greicius, 2010; Bullmore, 2012; Klöppel et al., 2012; Michel and Murray, 2012). While these approaches are promising, substantial obstacles remain that can drastically hinder the pace of progress if left unaddressed (Kelly et al., 2012).

In particular, the availability of large-scale imaging data is of paramount importance to the advancement of human brain imaging in neuropsychiatry (Van Horn and Gazzaniga, 2002; Buckner, 2010; Yeo et al., 2011; Milham, 2012). Myriad hypotheses exist regarding the etiology and manifestations of pathologic processes in the brain. It is only through the acquisition of large-scale imaging data with appropriate phenotyping (Bilder et al., 2009a,b; Cohen et al., 2011) that these hypotheses can be properly evaluated. Simultaneously, such datasets are a prerequisite to the deployment of discovery science approaches, which have the potential to yield more precise and empirically grounded hypotheses. Unfortunately, datasets of the prescribed scale are unprecedented in the imaging community, and particularly challenging for psychiatric imaging given its burdens (e.g., extensive time and substantial costs of recruitment, psychiatric assessment, and phenotyping). Individuals affected by psychiatric illness, as well as children, are also prone to a higher frequency of data loss due to motion (Power et al., 2012; Satterthwaite et al., 2012; Van Dijk et al., 2012; Wilke, 2012) and inability to tolerate the scanner environment, which only exacerbate the difficulties.

Fortunately, the 1000 Functional Connectomes Project (FCP) provided a model through which large-scale datasets can be obtained (Biswal et al., 2010; Milham, 2012). Specifically, the FCP pooled previously collected data from independent sites around the world, and demonstrated that discovery science could be performed on the aggregate sample. The FCP model of open sharing for the purposes of hypothesis testing and generation was not new, as a number of like minded efforts attempted sharing in the past (Van Horn et al., 2001; Marcus et al., 2007b; Weiner et al., 2012). Arguably, the FCP capitalized on the greater ease of sharing structural and resting state functional MRI datasets, whose methods are more amenable to sharing than task-based datasets. In addition, it highlighted the increasing willingness of many laboratories to participate in open science. Still, the FCP's success only represents an initial step in the implementation of open sharing in the imaging community as it only included non-clinical samples with phenotypes limited to age and sex.

Building on this model, functional neuroimaging investigators working on Attention-Deficit Hyperactivity Disorder (ADHD) in three continents came together to form the ADHD-200 Consortium (see Acknowledgments for ADHD-200 Consortium details). The effort was to establish a large-scale, aggregate resting state fMRI dataset, along with accompanying anatomical and phenotypic data for children and adolescents with ADHD. The consortium publicly released 776 resting state fMRI and anatomical datasets collected at eight independent imaging sites on March 1, 2011^1^ (Table 1). Included were 491 datasets obtained from typically developing individuals and 285 from children and adolescents diagnosed with ADHD, all between the ages of 7–21 years. The release was coordinated through the International Neuroimaging Data sharing Initiative (INDI^2^), which makes use of the web infrastructure provided by Neuroimaging Informatics Tools and Resources Clearinghouse (NITRC) NITRC.org. Accompanying phenotypic information includes: diagnostic status, dimensional ADHD symptom measures, age, sex, intelligence quotient (IQ), and lifetime medication status. Additionally, preliminary quality control assessments (usable vs. questionable) based upon visual time-series inspection were included for all resting state fMRI scans. The ADHD-200 release data are stored and distributed in two ways: via NITRC Resources (NITRC-R) as tarballs, and via NITRC Image Repository (NITRC-IR^3^) which supports searches by phenotypic information powered by XNAT (Marcus et al., 2007a).

**Table 1:** **Contributing sites**.

Contributing sites	Investigators	Age-range	TDC	ADHD
Brown University	Daniel P. Dickstein	8.5–17.8	27	24
Kennedy Krieger Institute	Stewart K. Mostofsky	8.3–11.8	61	22
New York University Langone Medical Center	F. Xavier Castellanos, Michael P. Milham, Adriana Di Martino, Clare Kelly, Maarten Mennes	7.1–17.9	99	123
NeuroImage	J. K. Buitelaar, J. A. Sergeant, R. B. Minderaa, A. Arias Vasquéz, S. V. Faraone, B. Franke, C. Hartman, D. Heslenfeld, P. Hoekstra, M. Luman, J. Oosterlaan, N. N. J. Rommelse, M. Zwiers	11–21.7	23	25
Peking University	Yu-feng Wang, Yu-fengZang, Li Sun, Qing-jiu Cao, Li An	8.4–17.3	146	113
Pittsburgh University	Beatriz Luna, Katerina Velanova, Miya Asato	10.1–20.4	95	6
Oregon Health and Sciences University	Damien Fair, Joel Nigg, Bonnie Nagel, Deepti Bathula, Swathi Iyer, Kathryn Mills, Taciana G. Costa Dias	7.1–11.9	42	37
Washington University-St. Louis	Bradley L. Schlaggar, Steve Petersen, Rebecca S. Coalson, Alecia C. Vogel, Jessica A. Church	7–21.8	61	0

*Bradley Hospital and Brown University were supported by NIMH K22/NIH recovery act supplement (3K22MH074945-02S1) and Bradley Hospital. The Kennedy Krieger Institute was supported by the Autism Speaks Foundation and NIH (R01 NS048527, R01MH078160 and R01MH085328), Johns Hopkins General Clinical Research Center (M01 RR00052), the National Center for Resource (P41 RR15241), and the Intellectual and Developmental Disabilities Research Center (HD-24061). NeuroImage was funded by NWO-groot (http://www.nwo.nl/projecten.nsf/pages/2300149796). The New York University Child Study Center was supported by the NIMH (R01MH083246), Autism Speaks Foundation, Stavros Niarchos Foundation, Leon Levy Foundation, and by an endowment from Phyllis Green and Randolph Cōwen. Oregon Health and Science University was supported by K99/R00 MH091238 (Fair), R01 MH086654 (Nigg), Oregon Clinical and Translational Research Institute (Fair), Medical Research Foundation (Fair), UNCF/Merck (Fair), and the Ford Foundation (Fair). Peking University was supported by the Commonwealth Sciences Foundation, Ministry of Health, China (200802073), National Foundation, Ministry of Science and Technology of China (2007BAI17B03), National Natural Sciences Foundation of China (30970802), Funds for International Cooperation of the National Natural Science Foundation of China (81020108022), National Natural Science Foundation of China (8100059), and Open Research Fund of the State Key Laboratory of Cognitive Neuroscience and Learning. The University of Pittsburgh was supported by Cognitive and Brain Systems Maturation (5R01 MH067924, Luna), Reward Processing in Adolescence (1R01 MH080243, Luna), Functional Anatomy of Adolescent ADHD: Defining markers of recovery (K01MH82123, Velanova). The Washington University-St. Louis was supported by the Brooks Family Fund, R01 HD057076 (Schlaggar), R01 NS046424, NIH NINDS NRSA (Church), NIH NIMH R21 (Schlaggar), TSA (Schlaggar), and TSA (Church). NITRC-R and NITRC-IR was supported by the National Institute of Biomedical Imaging and Bioengineering, National Institutes of Health, and Department of Health and Human Services (GSA Contract No. GS-00F-0034P, Order Number HHSN268200100090U, SBIR No. 1 R43 NS074540-01)*.

In sharing these data, the consortium realized the importance of reaching beyond the imaging community, which typically consists of psychiatrists, neurologists, and neuroscientists, to broader multidisciplinary scientific disciplines. To recruit the global scientific community to address childhood psychiatric illness, a competition was announced, with the goals of developing: (1) novel strategies for predicting diagnostic status based on an individual's intrinsic functional architecture and brain structure, and (2) novel techniques for identifying brain features that may yield ADHD biomarkers.

For the purposes of the competition, an additional 197 datasets from six imaging sites were released on July 1, 2011 without diagnostic labels (two of the six sites were not represented in the training set, augmenting the challenge). Fifty teams from around the world, representing a diverse array of backgrounds (e.g., mathematics, statistics, computer science, neuroscience) communicated their intent to compete, eventually yielding 21 submissions. This effort demonstrated the latent interest of the larger scientific community to develop effective prediction methodologies for psychiatric neuroimaging. Additionally, it encouraged additional open neuroscience efforts, such as the ADHD-200 Pre-Processed Initiative by the Neuro Bureau^4^, which provided pre-processed data to the broader community so as to bypass technical obstacles to wider participation.

In the current issue, several of the teams that participated in the ADHD-200 competition describe their techniques and results. These descriptions will provide the reader with insight into each team's decision-making process as they developed optimal diagnosis predictions in novel datasets. It is our hope that access to each team's methodology will spark new ideas and collaborations.

## Competition Results

The winning team for predicting diagnosis was from Johns Hopkins University, and included Brian Caffo, Ciprian Crainiceanu, AniEloyan, Fang Han, Han Liu, John Muschelli, Mary Beth Nebel, and Tuo Zhao. The Hopkins team scored 119 out of 195 points, with one point awarded per correct diagnosis (typically developing, ADHD primarily inattentive type, or ADHD combined type). A half point was awarded for a correct diagnosis of ADHD if the subtype was incorrect.

**Figure 1:**
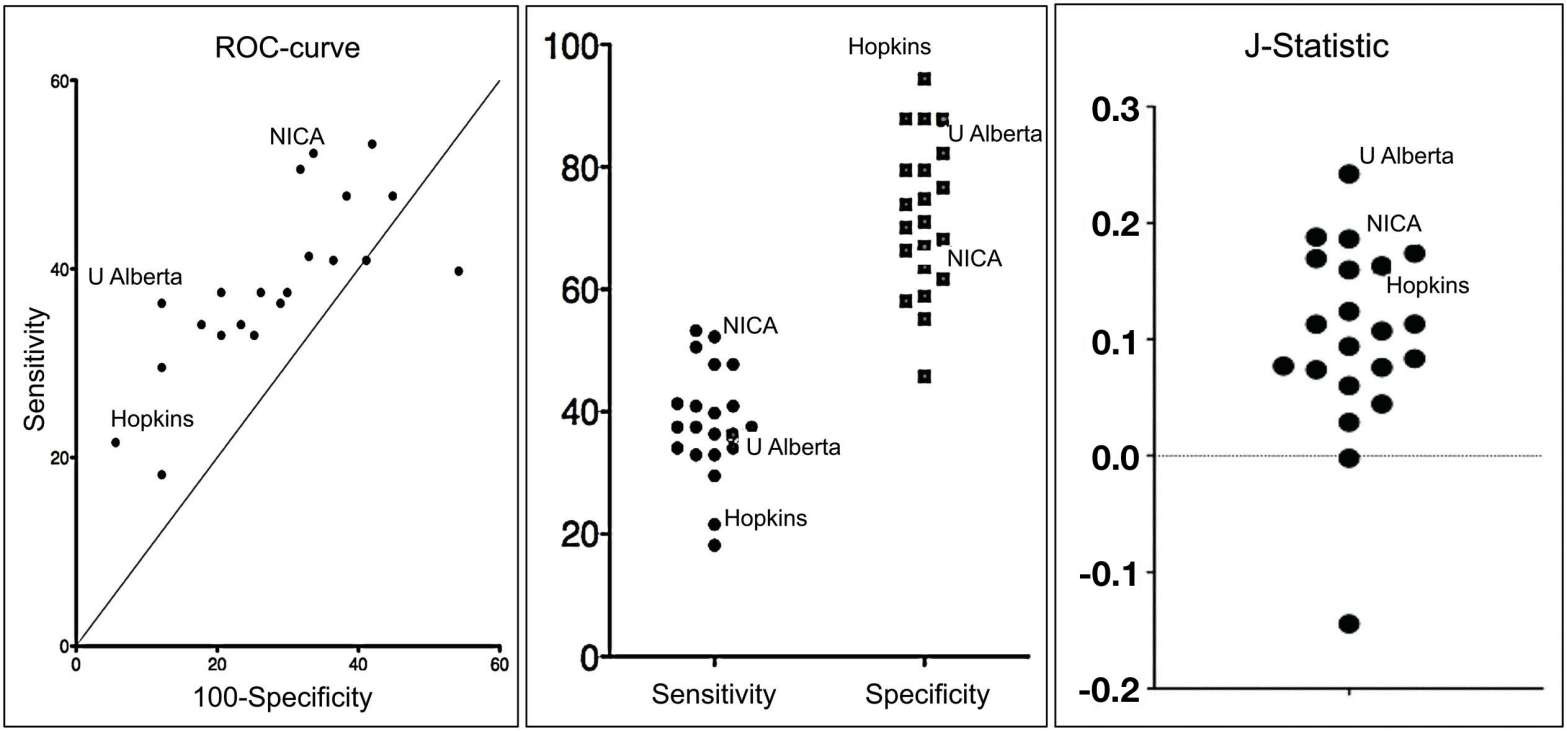
**Graphs depicting a receiver operating characteristic curve, comparison of sensitivity and specificity, and *J*-Statistic (calculated as sensitivity + specificity −1 and is thus a combination measure of sensitivity and specificity) for each team's solution**.

The method developed by the Hopkins team excelled in specificity, i.e., the ability to identify typically developing children (TDC) without falsely classifying them as having ADHD (see Figure 1).

They correctly classified 94% of TDC, showing that a diagnostic imaging methodology can be developed with a very low risk of false positives, a fantastic result. Their method was much less effective insensitivity, or its ability to identify true positive ADHD diagnoses. They only identified 21% of the clinically identified cases. However, among the cases they did capture, they discerned the correct ADHD subtype with 89.5% accuracy.

Other teams obtained substantially higher sensitivity scores. The methods developed by teams from the Chinese Academy of Sciences and the University of North Carolina at Chapel Hill both scored well on the *J*-statistic, a joint measure of specificity and sensitivity, suggesting that tests can be developed that can optimize both specificity and specificity (see Figure 1).

Prediction of diagnosis at chance levels would have yielded values between 33 and 38.75%. Participants’ predictions improved on chance by a healthy margin. The average prediction accuracy was 49.8% (range: 37.4–60.5%; 54.1% for datasets from sites included in the training set; 40.2% for datasets from sites not included in the training set).

Participants developed predictive methods that performed significantly above chance for analyzing datasets that were aggregated from multiple centers without prior coordination. These results suggest that progress toward developing effective predictive methods is possible even in less-than-ideal poorly controlled environments. We expect that these results will guide the psychiatric neuroimaging field as it grows. Despite the success of the methods developed in this competition, further development is necessary before the methods can be used in a clinical setting.

The winner in the biomarker contest was Che-Wei Chang from National Taiwan University, who brought emerging analytic approaches in computer vision to the study of ADHD-related differences in brain morphometry. By capturing novel aspects of brain anatomy, this effort defined a new feature upon which brain differences can be characterized and classified.

Intriguingly, the team from the University of Alberta consisting of Gagan Sidhu, Matthew Brown, Russell Greiner, Nasimeh Asgarian, and Meysam Bastani, did not use imaging data for their prediction model, but rather only phenotypic data of age, sex, handedness, and IQ. While this strategy was not consistent with the intended competition rules, the effort did garner the highest score, 124, and the highest prediction accuracy (62.5%).

The question regarding whether demographic features are better predictors of ADHD than imaging-based features naturally became a point of discussion in the imaging community^5^.

In short, the Alberta team's results drew attention to a major challenge faced by the field in the effort to generate predictive tools using existing data – group differences in base phenotypic variables that reflect population characteristics. ADHD in clinically referred samples is much more frequently recognized in boys than girls. As such, all studies have discrepant M:F ratios for ADHD and TDC groups. In the ADHD-200 sample, this discrepancy was the case (% males in the training set: TDC, 53%; ADHD, 79%; in the test set: TDC, 48%; ADHD, 71%). Similarly, performance IQ in ADHD is lower on average by 7–10 points than that of comparisons. In the ADHD-200 sample, IQ estimates differed between the TDC and ADHD groups (Training Set: 114 for TDC vs. 106 for ADHD, *p* < 0.001; Test Set: 113 for TDC vs. 103 for ADHD, *p* < 0.001). These baseline demographic/clinical differences clearly provided sufficient statistical power in the naturalistic/artificial context of a contest to yield substantial predictive power. In the real world, there are more than two options (ADHD, not ADHD) so that factors such as sex and IQ are inadequate for predicting diagnosis. In situations with limited options, such clues can be usefully exploited – as the Alberta group clearly demonstrated. Russ Poldrack has highlighted an even more significant concern in Tal Yarkoni's blog – what if imaging-based approaches are detecting the neural correlates of these phenotypic variables rather than neural correlates of the disorder itself? When we characterize differences in these populations, demographic factors should be considered, as they are for other diagnostic tests. Additionally, we recommend more careful consideration of such variables in the design of future training and test datasets.

## Concluding Remarks

We begin our concluding remarks by emphasizing that the consortium recognizes that diagnostic assessment cannot currently be based on structural or functional brain imaging, nor do we believe that brain imaging will ultimately result in a first-line tool in clinical psychiatry. The costs of conducting brain imaging for all patients who present with a potential neuropsychiatric disorder would be prohibitive. However, future brain imaging methods will likely have a role in diagnostic clarification, guiding treatment selection, and/or obtaining objective measures of treatment response. In other words, despite substantial costs, MRI could 1 day attain a reasonable level of utility for complex cases. In addition, it is probable that insights gained from exploration of MRI for diagnostic utility will be translatable into more readily available and cost effective tools, such as EEG or near-infrared spectroscopy. Importantly, predictive approaches also have the potential to inform our understanding of the neurobiological basis for ADHD by highlighting the findings that fared best as predictors.

We note that the primary goal of the ADHD-200 competition was to promote an open science model to foster competitive collaboration among members of the imaging community. The effort also aimed at encouraging the broader scientific community to join us in confronting the challenges of translational psychiatric imaging research. In this regard, this initial effort can already be characterized as a success and the efforts have engendered some momentum for the field. However, the members of the consortium acknowledge that this *post hoc*, uncoordinated dataset was not optimal for moving forward. For this advance to be accomplished, we estimate that a large-scale (e.g., 1000+) multimodal imaging dataset (e.g., resting state fMRI, diffusion tensor imaging, high-resolution structural scans) would need to be created and distributed to the scientific community. Unlike the ADHD-200 dataset that incorporated previously collected data, future reference datasets will require coordination of recruitment and phenotyping strategies. While we recognize the costs, large-scale, coordinated multicenter designs, such as the Alzheimer's Disease Neuroimaging Initiative and the Human Connectome Project, are ideal for such endeavors.

At a smaller, more feasible scale (and not mutually exclusive), would be efforts to harmonize data collection across independent imaging sites through the use of a core phenotypic protocol and open sharing of ADHD datasets along with core protocol-driven measures. Such a core phenotypic protocol could be as limited as that used by the ADHD-200, or more comprehensive with a brief cognitive battery (which would be preferable). Researchers would have the option not to disclose data beyond the core protocol. To accomplish this harmonized approach, community support for the establishment and coordination of a common phenotypic protocol for ADHD would be required, although even this approach would incur some costs.

While it is still unknown where open data sharing in psychiatric imaging will lead, it is clear that the field must transition from the current model into one that promotes coordinated, transparent, and open data sharing across laboratories and institutions. The costs of such an effort will not be small, but are dwarfed by the marked impairment and suffering in the lives of millions associated with disorders such as ADHD; failure to invest in such efforts will continue to cost much more in the long run and limit our ability to improve outcomes for those afflicted with neuropsychiatric disorders.
